# Maternal age and offspring developmental vulnerability at age five: A population-based cohort study of Australian children

**DOI:** 10.1371/journal.pmed.1002558

**Published:** 2018-04-24

**Authors:** Kathleen Falster, Mark Hanly, Emily Banks, John Lynch, Georgina Chambers, Marni Brownell, Sandra Eades, Louisa Jorm

**Affiliations:** 1 Centre for Big Data Research in Health, University of New South Wales, Sydney, Australia; 2 National Centre for Epidemiology and Population Health, Australian National University, Canberra, Australia; 3 Centre for Social Research Methods, Australian National University, Canberra, Australia; 4 The Sax Institute, Sydney, Australia; 5 School of Population Health, University of Adelaide, Adelaide, Australia; 6 School of Social and Community Medicine, University of Bristol, Bristol, United Kingdom; 7 Manitoba Centre for Health Policy, University of Manitoba, Winnipeg, Manitoba, Canada; 8 Baker IDI Heart and Diabetes Institute, Melbourne, Australia; University of Manchester, UNITED KINGDOM

## Abstract

**Background:**

In recent decades, there has been a shift to later childbearing in high-income countries. There is limited large-scale evidence of the relationship between maternal age and child outcomes beyond the perinatal period. The objective of this study is to quantify a child’s risk of developmental vulnerability at age five, according to their mother’s age at childbirth.

**Methods and findings:**

Linkage of population-level perinatal, hospital, and birth registration datasets to data from the Australian Early Development Census (AEDC) and school enrolments in Australia’s most populous state, New South Wales (NSW), enabled us to follow a cohort of 99,530 children from birth to their first year of school in 2009 or 2012. The study outcome was teacher-reported child development on five domains measured by the AEDC, including physical health and well-being, emotional maturity, social competence, language and cognitive skills, and communication skills and general knowledge. Developmental vulnerability was defined as domain scores below the 2009 AEDC 10th percentile cut point.

The mean maternal age at childbirth was 29.6 years (standard deviation [SD], 5.7), with 4,382 children (4.4%) born to mothers aged <20 years and 20,026 children (20.1%) born to mothers aged ≥35 years. The proportion vulnerable on ≥1 domains was 21% overall and followed a reverse J-shaped distribution according to maternal age: it was highest in children born to mothers aged ≤15 years, at 40% (95% CI, 32–49), and was lowest in children born to mothers aged between 30 years and ≤35 years, at 17%–18%. For maternal ages 36 years to ≥45 years, the proportion vulnerable on ≥1 domains increased to 17%–24%. Adjustment for sociodemographic characteristics significantly attenuated vulnerability risk in children born to younger mothers, while adjustment for potentially modifiable factors, such as antenatal visits, had little additional impact across all ages. Although the multi-agency linkage yielded a broad range of sociodemographic, perinatal, health, and developmental variables at the child’s birth and school entry, the study was necessarily limited to variables available in the source data, which were mostly recorded for administrative purposes.

**Conclusions:**

Increasing maternal age was associated with a lesser risk of developmental vulnerability for children born to mothers aged 15 years to about 30 years. In contrast, increasing maternal age beyond 35 years was generally associated with increasing vulnerability, broadly equivalent to the risk for children born to mothers in their early twenties, which is highly relevant in the international context of later childbearing. That socioeconomic disadvantage explained approximately half of the increased risk of developmental vulnerability associated with younger motherhood suggests there may be scope to improve population-level child development through policies and programs that support disadvantaged mothers and children.

## Abstract 2

In a population-based cohort study of Australian children, Kathleen Falster and colleagues examine the associations between maternal age and developmental outcomes in children at age five.

## Author summary

**Why was this study done?:**

- There has been a shift towards later childbearing in recent decades; although the perinatal risks are well documented, the consequences on child development are less clear.
- Several observational cohort studies have reported that increasing maternal age is associated with better child development outcomes, although the numbers of children born to older mothers were limited and broad maternal age categories have often been used.
- Recent evidence suggests that the relationship between older maternal age at childbirth and child cognitive ability may have shifted over time; this is largely explained by shifts in the sociodemographic and perinatal characteristics related to maternal age at childbirth.

**What did the researchers do and find?:**

- We used linked, population-level data to study the association between maternal age at childbirth and developmental vulnerability at age five in 99,530 children who started school in New South Wales, Australia, in 2009 or 2012.
- Children born to the youngest mothers had the highest risk of developmental vulnerability on all domains at age five, and the risk declined with increasing maternal age through to 30 years.
- Older motherhood was associated with a small increased risk of developmental vulnerability at age five, particularly in the physical health and well-being, social competence, and emotional maturity domains, equivalent to the risk for children born to mothers in their early twenties.
- Socioeconomic disadvantage largely accounted for the increased risk of developmental vulnerability associated with younger motherhood.

**What do these findings mean?:**

- To our knowledge, this study is the largest-scale evidence internationally on the relationship between maternal age at childbirth—across the whole distribution of maternal ages—and early childhood development.
- There may be scope to improve child development at a population level via policies and programs that support disadvantaged mothers and children.
- The small increased risk of developmental vulnerability in children born to older mothers is highly relevant in the international context of childbearing at increasingly older ages.
- Further research is needed to understand the mechanisms that underlie the elevated risk of developmental vulnerability in children born to older mothers, as well as the early childhood factors that potentially offset the increased pregnancy/birth risks associated with older motherhood.

## Introduction

In recent decades, there has been a shift towards later average age at childbearing in high-income countries, underpinned by an increasing proportion of women giving birth at older ages, combined with a reduction in teenage pregnancies [1]. Some of the primary drivers behind this trend have altered the age-related demographic profile of mothers over time. Whereas older mothers in previous generations often had higher parity and lower socioeconomic position, today it is common for mothers aged 35 years and older (henceforth ‘older mothers’) to be highly educated, professionally employed, and primigravida [2,3]. Because a woman’s childbearing age is related to biological, social, economic, and behavioural factors that may impact a child’s development from conception through childhood [2,4] and child development relates to later health and well-being [5,6], understanding the relationship between maternal age at childbirth and child development is important.

Although the increased perinatal risks of childbearing at both younger and older maternal ages are well documented [7–11] and the development of the offspring of younger mothers has received attention [12–16], few studies have examined child development across the full maternal age range; hence, the consequences of later childbearing on offspring development remain unclear [4]. Increasing maternal age has been associated with better cognitive ability [17], fewer social and emotional difficulties [18], and better language acquisition [18] in two studies of approximately 30,000 children, after accounting for differences in demographic and perinatal characteristics across the maternal age range. Several smaller cohort studies (<5,000 children) have also reported better development outcomes with increasing maternal age [19,20]; however, estimates were grouped into broad maternal age categories because of sample size constraints, masking potential variation in outcomes among children born to older mothers. In contrast, older maternal age (i.e., >35 years) was negatively associated with offspring cognitive ability measured in half a million men aged 17–20 years of age in Sweden [21]. The variation in conclusions between studies may be partially attributed to differences in sample size, study period, and the outcomes examined. For example, recent evidence suggests that cognitive development may have improved among children born to older mothers over time, largely accounted for by differences in socioeconomic and perinatal characteristics of mothers and infants between cohorts [3]. To provide a more accurate and policy-relevant picture of how offspring developmental outcomes vary across the maternal age range, including children born to mothers aged 35 years and older, larger sample sizes and contemporary estimates across a broad range of developmental domains are needed.

In this study, our objective was to quantify a child’s risk of developmental vulnerability on five domains—physical health and well-being, emotional maturity, social competence, language and cognitive skills, and communication skills and general knowledge—according to the mother’s age at childbirth, for the whole distribution of maternal ages in a contemporary cohort of 99,530 Australian children in their first year of school.

## Methods

This retrospective cohort study using linked, cross-sectoral population datasets is reported as per the RECORD guidelines [22] (S1 RECORD Checklist).

### Data sources, data linkage, and linked data resource

This study used data from the Australian Early Development Census (AEDC), which is a triennial, nationwide census of child development conducted since 2009 among children enrolled in the first year of full-time school [23]. In Australia’s most populous state, New South Wales (NSW), 97% of children enrolled in the first year of school participated in the 2009 and 2012 AEDC. A third-party agency (the NSW Centre for Health Record Linkage) linked the AEDC data to other population datasets in NSW, including the following used in this study: the Perinatal Data Collection; the Register of Births, Deaths and Marriages birth registrations; the Admitted Patient Data Collection; and Public School Enrolment records. Detailed information about the data sources, data linkage, and the population-based cohort of children included in the linked data resource for the broader ‘Seeding Success’ study have been reported elsewhere [24,25]. Briefly, the Seeding Success data resource includes data for a population-based cohort of children who were in their first year of school and had an AEDC record in 2009 or 2012 and a linked perinatal record and/or birth registration in NSW (*N* = 166,278 children). Of these, 7,755 (4.6%) children were identified as ‘high need requiring special assistance due to chronic medical, physical, or intellectually disabling conditions (e.g., Autism, Cerebral Palsy, Down Syndrome)’, based on teacher report of a medical diagnosis on the AEDC [26]. The AEDC scores of children with special needs were not included in the central derivation of national cut points for developmental vulnerability because the instrument had not been validated in children with special needs at that time.

### Study population for analysis

The study population for this analysis were selected from the 166,278 children in the Seeding Success data resource. For this analysis, we restricted the study population to (i) children enrolled in a NSW public school (*N* = 107,666), because parental education and occupation information are collected and available from public school enrolment records; (ii) singletons (*N* = 104,491), because of the greater risk of adverse perinatal and childhood outcomes in multiple gestation pregnancies and births; (iii) children with complete data for maternal age at childbirth and at least one outcome variable (*N* = 104,200); and (iv) children without special needs (*N* = 99,530), because the AEDC categorical outcome data were not available for children with special needs (S1 Fig).

### Child development outcomes

Early childhood development outcomes were measured using the AEDC, which collects teacher-reported information about a child’s development on the following domains: (1) physical health and well-being, (2) social competence, (3) emotional maturity, (4) language and cognitive skills, and (5) communication skills and general knowledge [23]. In Australia, the school year commences in late January/early February and the AEDC was conducted between May and August in 2009 and 2012. Because AEDC domain scores are highly skewed, we used the categorical AEDC outcomes for each domain, dichotomised into developmentally vulnerable or not. As per national reporting, the categorical outcomes, which are adjusted for the child’s year of age, classify children as developmentally vulnerable on each domain if they score below the 2009 AEDC 10th percentile cut point. Children were also classified as being developmentally vulnerable on one or more of the five domains. Several studies indicate acceptable measures of validity and reliability for the AEDC and its predecessor, the Canadian Early Development Instrument [27–32].

### Exposure

The month and year of birth for the mother and child were obtained from the birth registration, or the Perinatal Data Collection if the birth registration was unavailable, and used to calculate maternal age at childbirth, in years.

### Other analysis variables

We classified the following variables available in the source data as potential confounders: child’s age at the start of the school year, child’s sex, mother partnered/single parent at child’s birth, mother born in Australia or overseas, private health insurance/patient at child’s birth, number of previous pregnancies (i.e., parity), antenatal care before 20 weeks gestation, smoking during pregnancy, whether child speaks English as a second language, child’s Aboriginality (children were classified as Aboriginal and/or Torres Strait Islander if indicated for the child and/or either parent on the birth registration, perinatal, or hospital birth records or the AEDC [24]), AEDC year, mother’s highest level of school education, highest occupation level of either parent recorded on the child’s public school enrolment, geographical remoteness (defined by the Accessibility/Remoteness Index of Australia [ARIA+] [33]), and area-level socioeconomic disadvantage (defined by the Australian Bureau of Statistics’ Index of Relative Socio-economic Advantage and Disadvantage [34]). Area-level variables were assigned according to the mother’s statistical local area of residence at the child’s birth. A modifiable and potentially mediating variable available in the source data was participation in preschool/childcare in the year before school. Maternal age is a population risk indicator for the complex causal pathways associated with infant and childhood outcomes. We hypothesised that the following available variables may be on the causal pathway between maternal age at childbirth and child development at age five and, accordingly, did not adjust for these variables in the statistical models: gestational age, birth weight, preterm birth, small for gestational age, low 5-minute Apgar score, neonatal intensive care unit/special care nursery admission, resuscitation at birth, and additional developmental needs (e.g., hearing impairment).

### Missing data

For the 99,530 children included in the study population, the proportion of missing data for most covariates was <2%, with the exception of mother’s school education level (8.6%), the occupation level of either parent (6.9%), preschool/childcare (6.5%), mother single parent/partnered (3.1%), and antenatal care before 20 weeks gestation (2.3%) (S1 Table). In total, 20,833 children (20.9%) had missing data for one or more covariates. In response to peer review, imputation via chained equations [35] was used to generate five copies of the complete dataset with filled-in missing values, which we analysed in parallel, pooling estimates using standard rules [36] to optimise use of available data for the 99,530 children in the study population.

### Statistical analysis

Children were followed from birth until their first year of full-time school in 2009 or 2012. The distribution of maternal age at childbirth for children in the study population is presented as a histogram to illustrate the proportion of children contributing to the analysis at each maternal age. We estimated the proportion of children who were developmentally vulnerable on each domain, and ≥1 domains, with 95% confidence intervals, for every year of maternal age at childbirth; children born to mothers at the extremes of the maternal age range were grouped into ≤15 years and ≥45 years due to small numbers.

Our prespecified analysis plan involved fitting nonlinear regression models with a quadratic term to allow for the nonlinear relationship between maternal age and the risk of developmental vulnerability that we observed in the raw data. Following peer review, we applied piecewise linear regression models and compared results to those from the nonlinear regression models with the quadratic term (S1 Text). Based on the Akaike Information Criterion (AIC), there was a small—but statistically significant—improvement in the model fit using the piecewise linear regression methods, compared with the quadratic models (S2 Table). Analysis of residuals indicated that the improvement to model fit was primarily at the younger extreme of maternal age (S2 Fig). Although the substantive findings were similar using both methods (S3 Fig), we applied piecewise linear regression methods in the revised manuscript because of improved model fit.

Because of the small numbers of children born to mothers aged <15 years (*N* = 31) and >45 years (*N* = 67), the piecewise linear regression models were restricted to the 99,432 children in the study population who were born to mothers aged ≥15 to ≤45 years. For each year of maternal age at childbirth from 15 to 45 years, we estimated the absolute risk of developmental vulnerability on each domain, and ≥1 domain. The risk estimates are based on logistic regression models. However, instead of presenting the odds ratio estimates, we recycled the fitted model parameters to derive estimates of absolute risk using an approach referred to as regression risk modelling [37] or marginal standardisation [38]. The estimated model parameters from the logistic regression models were used to repeatedly calculate the probability of developmental vulnerability for each individual at hypothetical maternal ages from 15 to 45 years, conditional on their other observed model covariates. Averaging the resulting predictions across the whole population for a given maternal age returns an estimate of the risk of developmental vulnerability for that maternal age, assuming a common distribution of model covariates at each age. Maternal age was parameterised as a piecewise linear function with three segments: 15–<30 years, 30–<35 years, and 35–<45 years. The effect of maternal age was constrained to be zero between ages 30–<35. The choice of cut points was based on the pattern of risk observed in the raw data, commonly used maternal age groups in the related literature, and peer review. We also explored a parameterisation with four segments, which further divided the younger maternal age range into 15–<20 years and 20–<30 years; however, the improvement in model fit when specifying four segments compared to three did not compensate for the inclusion of an additional parameter; that is, there was no further reduction in the AIC between the two models (S2 Table). Accordingly, we selected the simpler model with maternal age parameterised as a piecewise linear function with three segments.

We fitted a sequence of regression models adjusted for child’s age at school entry, sex, and AEDC year (Model 1); we further adjusted for potential confounders, including private health insurance/patient, mother partnered/single parent, mother’s parity, mother born in Australia, whether child speaks English as a second language, child’s Aboriginality, highest level of maternal school education, highest occupation level of either parent, area-level socioeconomic disadvantage, and geographic remoteness (Model 2). We further adjusted for potentially modifiable factors, including antenatal care prior to 20 weeks gestation, smoking during pregnancy, and participation in preschool and/or childcare in the year before school (Model 3). Each model was estimated repeatedly on each of the five imputed datasets. Adjusted absolute risk estimates were calculated using the *adjrr* postestimation procedure [39]. Cluster-adjusted standard errors were applied in all models to account for the grouping of similar children within schools. The resulting estimates were combined using Rubin’s rules [36]. Analysis was conducted using Stata 12.1 [40].

### Sensitivity analyses

To assess the potential impact of excluding children from nongovernment schools, we compared the age- and sex-adjusted absolute risk estimates of developmental vulnerability on ≥1 domains in all children who had complete exposure data on the aggregate outcome available (*N* = 152,556) and the study population for this analysis, which was restricted to NSW public school children (*N* = 99,530). Because children with special needs lacked outcome data, and some of the conditions classified as special needs may be related to maternal age, we also conducted a sensitivity analysis that assumed a worst-case scenario, whereby all children with special needs were classed as developmentally vulnerable. To assess the difference between two approaches to addressing missing data, we compared the absolute risk estimates of developmental vulnerability on ≥1 domains from piecewise linear regression models applied to (i) the imputed data (*N* = 99,432) and (ii) the dataset restricted to children with complete covariate information (i.e., complete case data) (*N* = 78,293).

### Ethical approval

Ethical approval was obtained from the NSW Population Health Services and Research Ethics Committee (2014/04/523), the NSW Aboriginal Health and Medical Research Council Ethics Committee (1031/14), and the Australian National University Human Research Ethics Committee (2014/384), which included a waiver of consent to obtain the de-identified, population-level data for this record linkage study.

## Results

**Fig 1:**
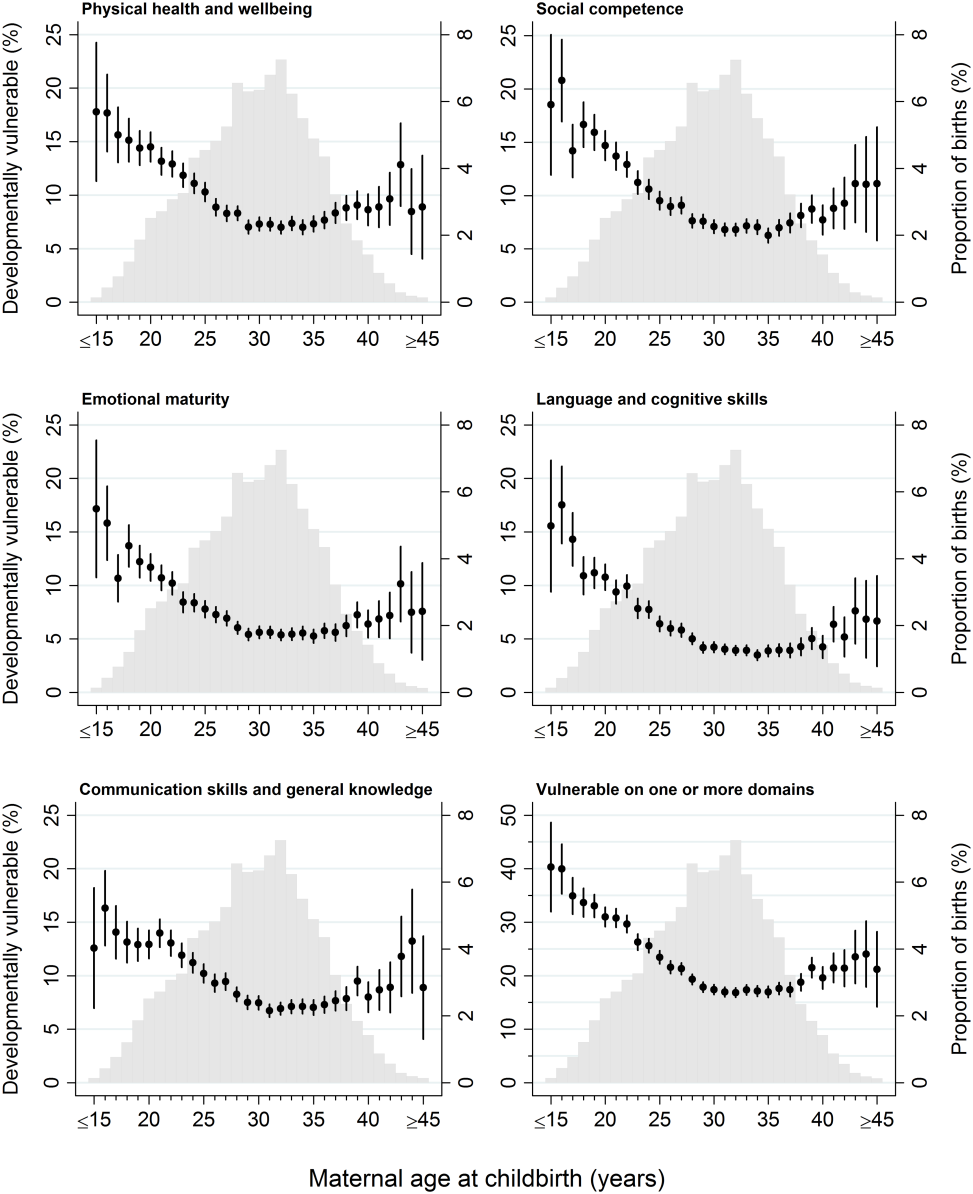
The distribution of maternal age at childbirth for children in the study population, overlaid with the proportion of children who were developmentally vulnerable on each outcome, by maternal age at childbirth. Light grey columns, study population birth distribution by year of maternal age at childbirth; black point estimates with 95% CIs, proportion of children developmentally vulnerable on each outcome (specified in figure subtitle).

The mean age of mothers when the study children were born was 29.6 years (standard deviation [SD], 5.7) and the median age was 30 years (interquartile range [IQR], 26–34; range, 13–56). The distribution of maternal age at childbirth for children in this study population peaked between 28 and 33 years; >6% of all children were born in each year of maternal age between 28 and 33 years, which, combined, equated to 40% of all children (Fig 1).

Children born to younger mothers were more likely to have indicators of socioeconomic disadvantage, including single parenthood, lower levels of maternal education, and parental occupation, whereas older mothers were more likely to have indicators of socioeconomic advantage, such as private health insurance and living in major cities and more socioeconomically advantaged areas (Table 1). It was more common for young mothers not to attend antenatal care before 20 weeks gestation and to smoke during pregnancy, compared with older mothers. Children born to younger mothers were less likely to attend preschool/day care in the year before school, although >80% of children born to mothers aged <20 years attended preschool/day care.

**Table 1:** Sociodemographic, pregnancy, and early childhood characteristics, by maternal age at childbirth, for the 99,530 children in the study population^1^.

	Maternal age at childbirth (years)										Total	
	<20		20–<25		25–<30		30–<35		≥35			
Total, *n* (%)	4,382	(100)	15,815	(100)	27,332	(100)	31,975	(100)	20,026	(100)	99,530	(100)
**Sociodemographic characteristics at child’s birth**												
Age at start of school (years), mean (SD)	5.2	(0.4)	5.2	(0.4)	5.2	(0.4)	5.2	(0.3)	5.2	(0.3)	5.2	(0.3)
Female sex, *n* (%)	2,199	(50.2)	7,736	(48.9)	13,483	(49.3)	15,713	(49.1)	9,909	(49.5)	49,040	(49.3)
Aboriginal and/or Torres Strait Islander^2^, *n* (%)	1,239	(28.3)	2,217	(14.0)	1,803	(6.6)	1,301	(4.1)	646	(3.2)	7,206	(7.2)
Mother born in Australia^3^, *n* (%)	3,979	(90.8)	12,755	(80.7)	20,490	(75.0)	22,866	(71.5)	12,689	(63.4)	72,779	(73.1)
Mother married/partnered^3^, *n* (%)	1,473	(33.6)	9,761	(61.7)	22,666	(82.9)	28,653	(89.6)	17,768	(88.7)	80,321	(80.7)
Private health insurance/patient^3^, *n* (%)	172	(3.9)	1,274	(8.1)	6,925	(25.3)	13,387	(41.9)	9,157	(45.7)	30,916	(31.1)
Lived in major city^4^, *n* (%)	2,022	(46.1)	8,741	(55.3)	16,805	(61.5)	21,760	(68.1)	14,390	(71.9)	63,718	(64.0)
Lived in inner regional area^4^, *n* (%)	1,550	(35.4)	4,740	(30.0)	7,685	(28.1)	7,632	(23.9)	4,221	(21.1)	25,829	(26.0)
Lived in outer regional area^4^, *n* (%)	692	(15.8)	2,086	(13.2)	2,569	(9.4)	2,358	(7.4)	1,308	(6.5)	9,012	(9.1)
Lived in remote/very remote area^4^, *n* (%)	117	(2.7)	248	(1.6)	273	(1.0)	226	(0.7)	107	(0.5)	971	(1.0)
Area-level disadvantage^4^, *n* (%)												
Quintile 1 (Most disadvantaged)	826	(18.8)	2,313	(14.6)	2,772	(10.1)	2,334	(7.3)	1,331	(6.6)	9,575	(9.6)
Quintile 2	763	(17.4)	2,610	(16.5)	3,240	(11.9)	2,899	(9.1)	1,659	(8.3)	11,171	(11.2)
Quintile 3	1,955	(44.6)	7,036	(44.5)	10,702	(39.2)	9,792	(30.6)	5,315	(26.5)	34,801	(35.0)
Quintile 4	631	(14.4)	2,701	(17.1)	5,948	(21.8)	7,087	(22.2)	4,137	(20.7)	20,504	(20.6)
Quintile 5 (Least disadvantaged)	207	(4.7)	1,154	(7.3)	4,669	(17.1)	9,864	(30.8)	7,584	(37.9)	23,479	(23.6)
**Pregnancy characteristics relating to child’s birth**												
Mother had no prior births, *n* (%)	3,617	(82.5)	8,461	(53.5)	12,063	(44.1)	11,309	(35.4)	5,240	(26.2)	40,691	(40.9)
Mother had one prior birth, *n* (%)	673	(15.4)	5,155	(32.6)	9,217	(33.7)	11,864	(37.1)	7,211	(36.0)	34,120	(34.3)
Mother had two or more prior births, *n* (%)	92	(2.1)	2,199	(13.9)	6,052	(22.1)	8,802	(27.5)	7,576	(37.8)	24,719	(24.8)
Antenatal care before 20 weeks gestation, *n* (%)	3,377	(77.1)	13,227	(83.6)	24,240	(88.7)	29,183	(91.3)	18,134	(90.6)	88,161	(88.6)
Mother smoked during pregnancy, *n* (%)	1,795	(41.0)	4,554	(28.8)	4,418	(16.2)	3,548	(11.1)	2,235	(11.2)	16,549	(16.6)
**Sociodemographic and other characteristics measured in child’s first year at school**												
AEDC year, 2009	2,156	(49.2)	7,659	(48.4)	13,229	(48.4)	15,240	(47.7)	8,799	(43.9)	47,083	(47.3)
AEDC year, 2012	2,226	(50.8)	8,156	(51.6)	14,103	(51.6)	16,735	(52.3)	11,227	(56.1)	52,447	(52.7)
Preschool/childcare in year before school, *n* (%)	3,591	(81.9)	13,448	(85.0)	24,176	(88.5)	28,834	(90.2)	17,835	(89.1)	87,885	(88.3)
English second language, *n* (%)	408	(9.3)	2,748	(17.4)	4,993	(18.3)	5,316	(16.6)	3,449	(17.2)	16,914	(17.0)
Mother completed Year 12^5^ at school, *n* (%)	1,030	(23.5)	7,248	(45.8)	17,684	(64.7)	22,390	(70.0)	13,143	(65.6)	61,494	(61.8)
Mother completed Year 11 at school, *n* (%)	634	(14.5)	1,897	(12.0)	2,206	(8.1)	1,790	(5.6)	1,053	(5.3)	7,580	(7.6)
Mother completed Year 10 at school, *n* (%)	1,879	(42.9)	5,064	(32.0)	5,894	(21.6)	6,419	(20.1)	4,684	(23.4)	23,940	(24.1)
Mother completed ≤Year 9 at school, *n* (%)	839	(19.1)	1,606	(10.2)	1,549	(5.7)	1,377	(4.3)	1,146	(5.7)	6,516	(6.5)
Highest level occupation of either parent^6^, *n* (%)												
Managers/professionals	178	(4.1)	1,472	(9.3)	5,766	(21.1)	10,298	(32.2)	6,949	(34.7)	24,662	(24.8)
Business managers/associate professionals	400	(9.1)	2,485	(15.7)	6,493	(23.8)	8,656	(27.1)	5,226	(26.1)	23,259	(23.4)
Trades/clerks/services	1,157	(26.4)	4,873	(30.8)	7,775	(28.4)	7,130	(22.3)	3,942	(19.7)	24,878	(25.0)
Drivers/hospitality/labourers	1,394	(31.8)	4,375	(27.7)	4,906	(17.9)	3,991	(12.5)	2,523	(12.6)	17,190	(17.3)
Not in paid work in last 12 months	1,253	(28.6)	2,609	(16.5)	2,392	(8.8)	1,901	(5.9)	1,386	(6.9)	9,541	(9.6)

^1^Based on imputed data for the 99,530 children in the study population.

^2^Defined as child or parent identified as Aboriginal or Torres Strait Islander on any of the birth records (i.e., Perinatal Data Collection, birth registration, or hospital birth record) or AEDC school record.

^3^Based on hospital birth record.

^4^Based on mother’s statistical local area of residence recorded in the Perinatal Data Collection.

^5^Highest level of school education in Australia

^6^Based on highest ranking occupation of either parent recorded on school enrolment.

Abbreviations: AEDC, Australian Early Development Census; SD, standard deviation.

Of the 99,530 children in the study population, outcome data were available for 99,437 children on the physical health and well-being domain, 99,380 children on the social competence domain, 98,935 children on the emotional maturity domain, 99,434 children on the language and cognitive skills domain, 99,428 children on the communication skills and general knowledge domain, and 99,015 children on the ‘vulnerable on ≥1 domain’ aggregate outcome (S1 Table). The proportion of children vulnerable on the five AEDC domains and vulnerable on ≥1 domain followed a reverse J-shaped distribution across the maternal age range (Fig 1). Children born to the youngest mothers had the highest proportion of developmentally vulnerable. Among children born to mothers aged ≤15 years at childbirth, 13%–19% were vulnerable on each of the five developmental domains and 40% (95% CI, 32%–49%) were vulnerable on ≥1 domains (Fig 1). The proportion vulnerable on each AEDC domain, and ≥1 domain, decreased with increasing maternal age up to 29–35 years. For children born to mothers aged 29–35 years of age, the proportion vulnerable for every year of maternal age was <5% on the language and cognitive domain, 4%–8% on the other four domains, and 17% (95% CI, 16%–18%) to 18% (95% CI, 17%–19%) on ≥1 domain. Among children born to mothers aged 36 years and older, the proportion vulnerable generally increased, ranging between 3% and 14% on the five AEDC domains and 17% (95% CI, 16%–19%) to 24% (95% CI, 18%–30%) vulnerable on ≥1 domain; however, the point estimates were variable and the confidence intervals wider because fewer children were born to mothers aged >35 years.

**Fig 2:**
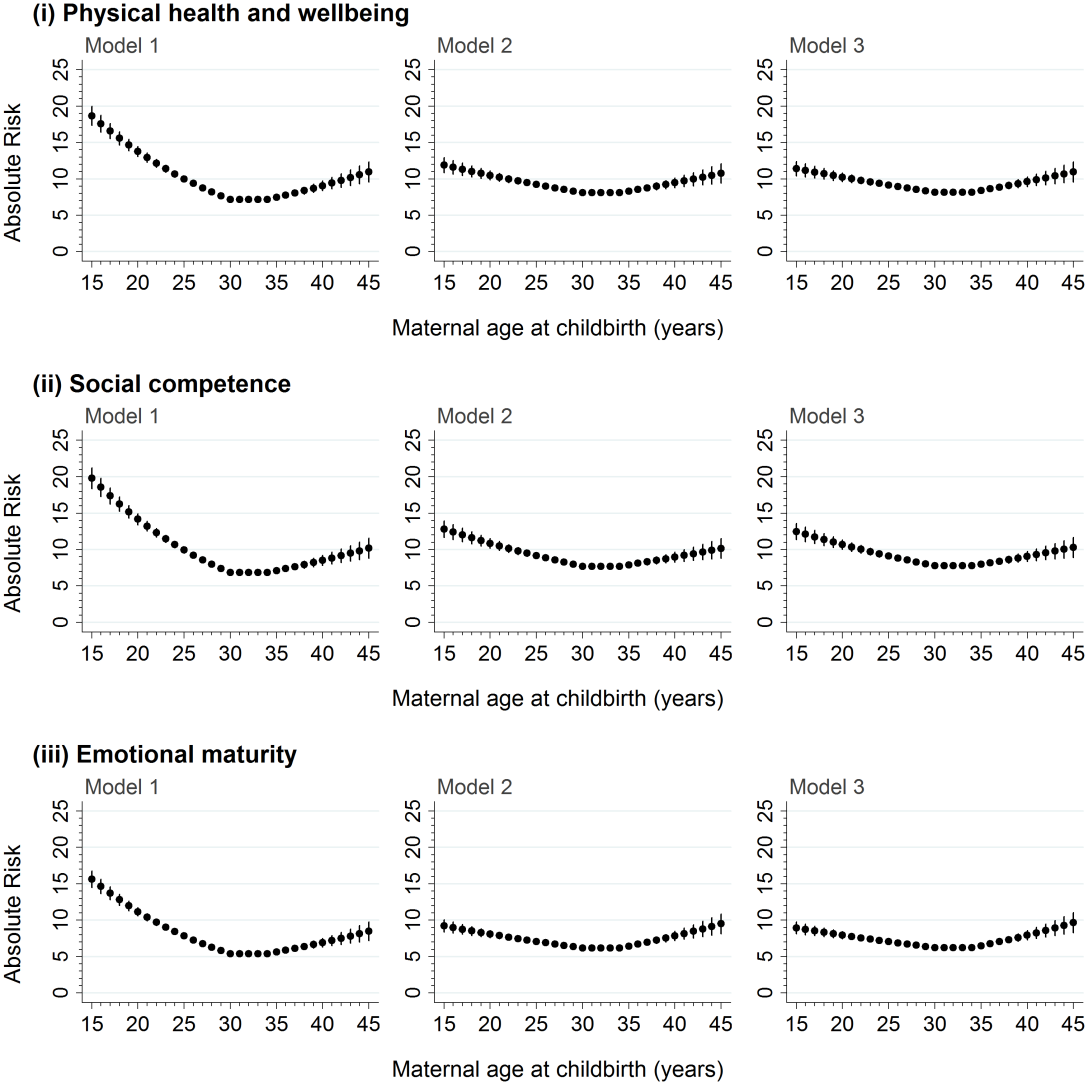
The absolute risk of developmental vulnerability on the physical health and well-being, social competence, and emotional maturity domains of the AEDC for every year of maternal age at childbirth between 15 and 45 years. Model 1 includes adjustment for the child’s age at school entry, sex, and AEDC year; in addition to the covariates included in Model 1, Model 2 adjusts for private health insurance/patient status, mother born in Australia/overseas, mother partnered/single parent, mother’s parity, child’s Aboriginality, whether child speaks English as a second language, highest level of maternal school education, highest level of occupation of either parent, area-level disadvantage, and geographical remoteness; in addition to the covariates included in Model 2, Model 3 adjusts for antenatal care visit before 20 weeks gestation, smoking during pregnancy, and preschool/day care attendance in the year before school. AEDC, Australian Early Development Census.

**Fig 3:**
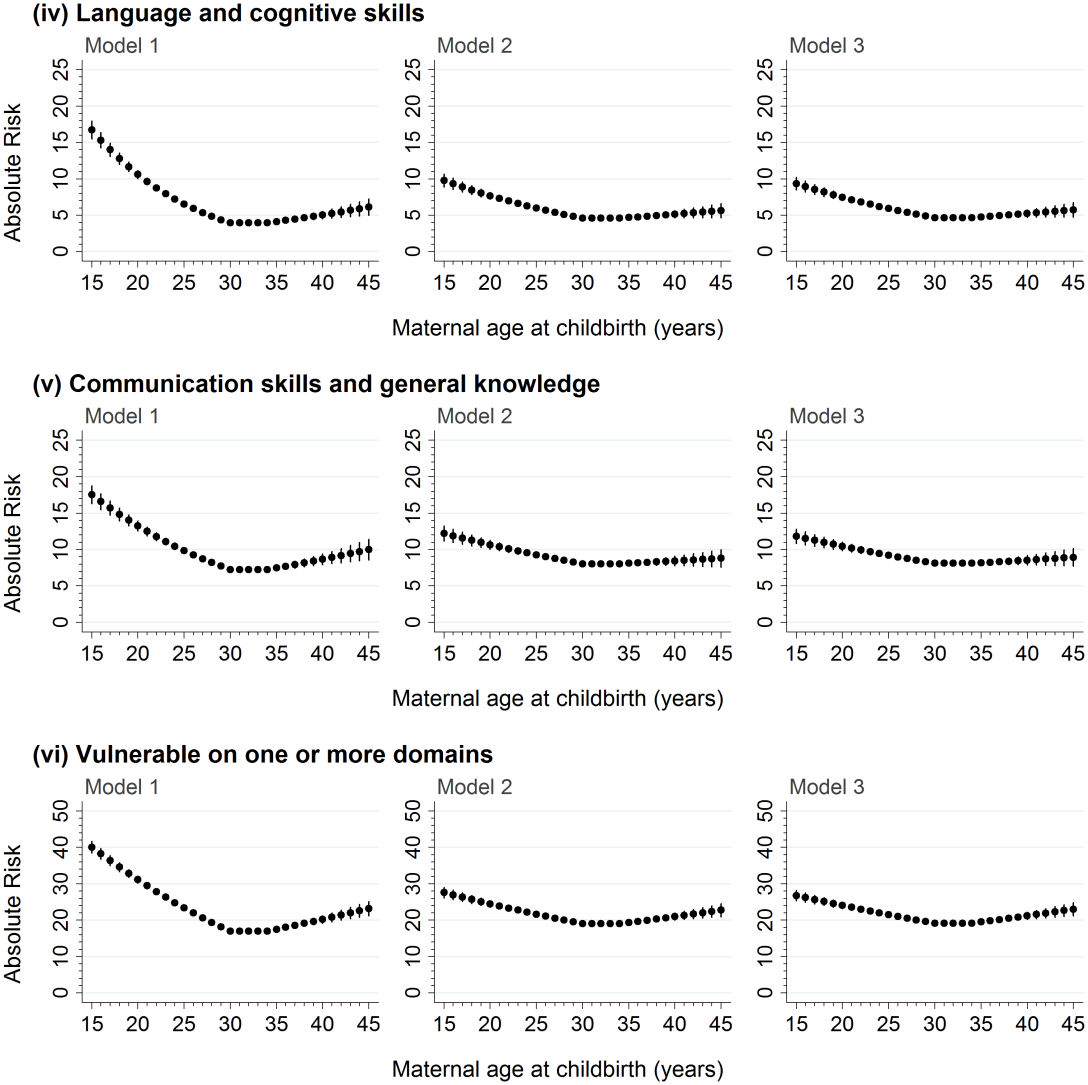
The absolute risk of developmental vulnerability on the language and cognitive skills and communication skills and general knowledge domains of the AEDC, and vulnerability on ≥1 AEDC domains, for every year of maternal age at childbirth between 15 and 45 years. Model 1 includes adjustment for the child’s age at school entry, sex, and AEDC year; in addition to the covariates included in Model 1, Model 2 adjusts for private health insurance/patient status, mother born in Australia/overseas, mother partnered/single parent, mother’s parity, child’s Aboriginality, whether child speaks English as a second language, highest level of maternal school education, highest level of occupation of either parent, area-level disadvantage, and geographical remoteness; in addition to the covariates included in Model 2, Model 3 adjusts for antenatal care visit before 20 weeks gestation, smoking during pregnancy, and preschool/day care attendance in the year before school. AEDC, Australian Early Development Census.

For the 99,432 children who were born to mothers aged ≥15–≤45 years, there was a significant negative association between maternal age and developmental vulnerability in the 15–<30-year maternal age range and a significant positive association in the ≥35–45 year maternal age range on the five AEDC domains, and ≥1 domain, in the models adjusted for age, sex, and year (Table 2; Model 1, *p* < 0.001 on all). Adjustment for potential confounders attenuated the association in the 15–<30-year maternal age range on all outcome measures, compared with Model 1, but had a smaller impact in the ≥35–45-year maternal age range (Table 2; Model 2). Further adjustment for potentially modifiable factors, such as antenatal care, smoking during pregnancy, and preschool/childcare, accounted for minimal additional differences in the association between maternal age and developmental vulnerability for all outcomes (Table 2, Model 3). The risk estimates presented in Figs 2 and 3 confirm this pattern. For example, the estimated risk of developmental vulnerability on ≥1 domains was 40.1% (95% CI, 38.5%–41.7%) for children born to mothers aged 15 years in Model 1 (adjusted for age, sex, and year), which was attenuated to 27.6% (95% CI, 26.2%–29.0%) after adjusting for sociodemographic and perinatal characteristics in Model 2, and 26.8% (95% CI, 25.4%–28.2%) after further adjusting for potentially modifiable factors in Model 3 (Fig 3). In contrast, the estimated risk was relatively stable across the three models in the ≥35–45-year maternal age range. For example, among children born to mothers aged 45 years, the adjusted risk of developmental vulnerability on ≥1 domain was 23.2% (95% CI, 21.3%–25.2%) in Model 1, 22.8% (95% CI, 21.0%–24.6%) in Model 2, and 23.0% (95% CI, 21.2%–24.8%) in Model 3 (Fig 3).

**Table 2:** Coefficients for 1-year increase in maternal age between 15–<30 years, and ≥35–45 years, from piecewise linear regression models.

	Maternal age at childbirth					
	15–<30 years			≥35–45 years		
	ß	95% CI	*p*-value	ß	95% CI	*p*-value
Physical health and well-being						
Model 1^a^	−0.074	(−0.081–−0.067)	<0.001	0.043	(0.029–0.057)	<0.001
Model 2^b^	−0.030	(−0.038–−0.022)	<0.001	0.030	(0.015–0.044)	<0.001
Model 3^c^	−0.026	(−0.034–−0.018)	<0.001	0.031	(0.017–0.045)	<0.001
Social competence						
Model 1^a^	−0.082	(−0.090–−0.075)	<0.001	0.040	(0.025–0.055)	<0.001
Model 2^b^	−0.039	(−0.047–−0.031)	<0.001	0.029	(0.014–0.044)	<0.001
Model 3^c^	−0.037	(−0.045–−0.029)	<0.001	0.029	(0.014–0.044)	<0.001
Emotional maturity						
Model 1^a^	−0.081	(−0.088–−0.074)	<0.001	0.045	(0.029–0.061)	<0.001
Model 2^b^	−0.030	(−0.038–−0.022)	<0.001	0.044	(0.028–0.060)	<0.001
Model 3^c^	−0.027	(−0.036–−0.019)	<0.001	0.045	(0.029–0.061)	<0.001
Language and cognitive skills						
Model 1^a^	−0.107	(−0.115–−0.099)	<0.001	0.042	(0.024–0.061)	<0.001
Model 2^b^	−0.058	(−0.067–−0.049)	<0.001	0.020	(0.001–0.039)	0.035
Model 3^c^	−0.054	(−0.063–−0.044)	<0.001	0.022	(0.002–0.041)	0.027
Communication skills and general knowledge						
Model 1^a^	−0.068	(−0.075–−0.060)	<0.001	0.032	(0.017–0.047)	<0.001
Model 2^b^	−0.034	(−0.042–−0.026)	<0.001	0.010	(−0.006–0.025)	0.222
Model 3^c^	−0.031	(−0.039–−0.023)	<0.001	0.010	(−0.005–0.026)	0.190
Vulnerable on one or more domains						
Model 1^a^	−0.082	(−0.087–−0.076)	<0.001	0.036	(0.026–0.047)	<0.001
Model 2^b^	−0.035	(−0.041–−0.029)	<0.001	0.023	(0.012–0.033)	<0.001
Model 3^c^	−0.032	(−0.038–−0.026)	<0.001	0.023	(0.012–0.034)	<0.001

^a^Model 1 includes adjustment for the child’s age at school entry, sex, and AEDC year.

^b^In addition to the covariates included in Model 1, Model 2 adjusts for private health insurance/patient status, mother born in Australia/overseas, mother partnered/single parent, mother’s parity, child’s Aboriginality, whether child speaks English as a second language, highest level of maternal school education, highest level of occupation of either parent, area-level disadvantage, and geographical remoteness.

^c^In addition to the covariates included in Model 2, Model 3 adjusts for antenatal care visit before 20 weeks gestation, smoking during pregnancy, and preschool/day care attendance in the year before school.

Abbreviation: AEDC, Australian Early Development Census.

Sensitivity analyses revealed that the overall association between maternal age at childbirth and developmental vulnerability at age five was not materially different between the sample of children with complete exposure information and at least one outcome and the study population who attended NSW public schools (S4 Fig). Moreover, the pattern of association between maternal age and developmental vulnerability was similar to the main analysis when children with special needs were included in the developmentally vulnerable group (S5 Fig) and when analyses were conducted on imputed versus complete case datasets (S6 Fig).

## Discussion

### Main findings

In this large, population-based cohort study, we found that the risk of developmental vulnerability on five domains followed a reverse J-shaped association according to maternal age at childbirth. The risk of developmental vulnerability was highest in children born to the youngest mothers and decreased with every additional year of maternal age through to the late twenties/early thirties, depending on the developmental domain. The risk of developmental vulnerability was lowest in children born to mothers in their late twenties to mid-thirties, corresponding with the maternal age range with the highest birth rates. Among children born to mothers older than 35 years, the risk of developmental vulnerability generally increased, although the risk estimates were variable and the confidence intervals wider due to the decreasing number of births at the older extreme of maternal age. Adjustment for sociodemographic characteristics accounted for a substantial proportion of the increased risk of developmental vulnerability associated with younger motherhood. On the measures of language and cognition, and communication skills and general knowledge, adjustment for sociodemographic characteristics accounted for most of the small but significant increased risk of developmental vulnerability associated with older motherhood. Further adjustment for modifiable factors—including antenatal care attendance, smoking during pregnancy, and preschool/childcare—accounted for minimal additional absolute differences in developmental vulnerability across the maternal age range.

### Strengths and limitations

The main strengths of this study were the large sample size and broad range of developmental outcomes collected on a contemporary general population. Limitations of this study potentially include bias, such as selection and measurement biases. However, derivation of the study population from linked population-level datasets minimised the selection biases associated with nonresponse and attrition in traditional cohort studies, and recall and social desirability biases were also minimised through the use of data recorded by midwives and teachers instead of self- or parent report. The multi-agency linkage yielded a broad range of sociodemographic, perinatal, health, and developmental variables at the child’s birth and school entry; however, we were necessarily limited to available data sources and variables available in the source data, which were recorded for administrative purposes. For example, school enrolment data were available for children enrolled in NSW public schools but not for children who were enrolled in nongovernment schools in NSW. Because school enrolment data contain information on parental education and occupation, we restricted our study population to the 65% of children enrolled in NSW public schools for this study. Although this reduced our sample size, sensitivity analyses suggest the relationship between maternal age and child development was similar in children attending government and nongovernment schools. Another limitation was incomplete information for several covariates, which was more common among children born to younger mothers. To optimise the use of available data, we imputed missing values for the main analysis, which produced slightly lower estimates of the risk of vulnerability at the younger extreme of maternal age compared with analysis of children with complete covariate information. Although we were able to adjust for several indicators of family-level socioeconomic disadvantage in this study, we cannot rule out residual confounding relating to unmeasured characteristics. Furthermore, we were unable to explore certain potential mediating factors (e.g., parenting behaviours) or the use of assisted reproduction using the available data. Another limitation was the lack of outcome data for and subsequent exclusion of children with special needs; this is potentially problematic because maternal age may be associated with some of the special needs conditions, including chromosomal and congenital anomalies [41–43]. However, our ‘worst-case scenario’ sensitivity analysis, whereby all children with special needs were classified as developmentally vulnerable, suggests that the overall study conclusions were not affected by the exclusion of children with special needs. In addition to chromosomal and congenital anomalies, maternal age is associated with termination of pregnancy [44] and the risk of perinatal mortality [11], and thus our study only evaluated children who survived to school age. Although our study adds to the literature on advanced maternal age as a population risk marker for some adverse offspring outcomes, further investigation into the role of specific pregnancy and obstetric factors (e.g., preterm birth, maternal hypertension, or diabetes) that lie on the causal pathway between maternal age and developmental vulnerability at age five is warranted.

### Comparison with other studies

Our finding that the risk of developmental vulnerability on all domains decreased with every additional year of maternal age between 15 and 30 years is consistent with several previous studies of childhood development [3,17,18] as well as adverse perinatal outcomes [7,9,45], psychosocial and behavioural problems [19,46,47], academic outcomes [13], and adult cognitive ability [21]. Meanwhile, our finding that there was a small increase in the risk of developmental vulnerability of children born to older mothers, equivalent to the risk for children born to mothers in their early twenties, suggests there may be limits to the previously claimed benefits of increasing maternal age on offspring childhood development [17,18,20]. One of the important factors that may underlie the varying conclusions between studies is difference in the scale of the evidence. To our knowledge, our study had more than double the sample size of the largest and most comparable previous studies of childhood development [17,18], including more than 16,000 children born to mothers aged 35 years and older. Accordingly, we were able to observe outcomes by year of maternal age through to 45 years, offering the highest-resolution picture of early childhood development at the older extreme of the maternal age range to date. Of note, the pattern of risk for early childhood developmental vulnerability in our study is consistent with the previously observed relationships between older maternal age and offspring academic outcomes at age 16 years [48] and offspring adult cognitive ability [21] from studies that followed more than half a million individuals using linked register data in Sweden. Another factor relevant to the comparison of findings between studies is whether the unadjusted association between maternal age and childhood development was reported in addition to the adjusted association. Because the two largest and most comparable previous studies of childhood development did not report the unadjusted estimates from statistical models [17,18], we are unable to compare the distribution of development outcomes across every year of the maternal age range and how the adjustment for potential confounders impacted on the risk estimates in each cohort. For this reason, it is not clear whether adjustment for socioeconomic disadvantage attenuated the risk in children born to younger mothers in previous studies, as observed in our study. Other important factors that may at least in part have contributed to the variation in findings between studies are the time period [3] and the type of outcomes measured. Outcome measures that quantify the average level of development rather than developmental vulnerability may have a different pattern of association with maternal age—for example, if there is greater variation in such measures at the older extreme of maternal age.

### Meaning of the findings and implications

Historically, there has been considerable focus on negative outcomes among offspring of young mothers, often defined as 20 years or less. Although these children experience the highest risk, our data illustrate there is a continuing decline in the risk of developmental vulnerability with increasing maternal age, from children born to the very youngest mothers through to mothers in their early thirties, and this is largely underpinned by disadvantage. Furthermore, few children are born to young mothers. In this context, policies and programs that target disadvantaged mothers and children rather than focusing on children born to young mothers alone are likely to reach more children at risk of poor development outcomes and have a greater impact on child development at a population level [12,49]. At the other end of the spectrum, it is well established that the offspring of older mothers have a greater risk of chromosomal abnormalities [10], congenital conditions [41], and adverse perinatal outcomes, including preterm birth and being small for gestational age [9], although prenatal screening has impacted the incidence of children born with chromosomal and congenital abnormalities [50]. Adverse perinatal outcomes are, in turn, associated with later child development [51,52]. It has been argued that, beyond the perinatal period, the socioeconomic and other potential advantages of older motherhood may offset the biological disadvantages during pregnancy and childbirth [53]. However, limitations in the scale of the evidence in previous studies may have oversimplified the pattern of risk of developmental vulnerability at the older extreme of maternal age. In the context of the later childbearing trend, even a small increase in the risk of developmental vulnerability among children born to older mothers may be of population-level importance in terms of later health and well-being [21].

### Conclusions

In this, the largest and most comprehensive study of early childhood development outcomes and maternal age to our knowledge, to date, we have confirmed that children born to younger mothers have the highest risk of developmental vulnerability. In addition, we identified a small increased risk of developmental vulnerability in children born to older mothers, which is highly relevant in the international context of childbearing at increasingly older ages. That the increased risk of developmental vulnerability among children born to the very youngest through to average-aged mothers was largely explained by socioeconomic disadvantage suggests there may be scope to improve child development at a population level through policies and programs that support disadvantaged mothers and children. Future research to elucidate the mechanisms that underlie the elevated risk of developmental vulnerability in children born to older mothers, as well as the early childhood factors (such as parenting behaviours) that potentially offset the increased perinatal risks associated with older motherhood, may further inform policies and interventions to promote positive child development across the population.

## Supporting information

S1 Fig Process for identifying the study population for analysis from the Seeding Success data resource. ^a^In 2010, additional AEDC data were collected in NSW to increase numbers of children in areas with small sample sizes in the 2009 AEDC. ^b^These children with missing outcome data (*n* = 286) did not include children with medically diagnosed special needs who were excluded in the following step. AEDC, Australian Early Development Census; NSW, New South Wales.
(TIF)

S2 Fig Residual plots from quadratic and piecewise linear regression models of developmental vulnerability on ≥1 AEDC domain, by maternal age at childbirth, using complete case data (*N* = 78,229). AEDC, Australian Early Development Census.
(TIF)

S3 Fig Comparison of results from quadratic versus piecewise linear regression models using complete case data (*N* = 78,229) to estimate the absolute risk of developmental vulnerability on ≥1 AEDC domain, by maternal age at childbirth. Reference group for quadratic models, maternal age at childbirth of 30 years. Model 1 includes adjustment for the child’s age at school entry, sex, and AEDC year; in addition to the covariates included in Model 1, Model 3 adjusts for private health insurance/patient status, mother born in Australia/overseas, mother partnered/single parent, mother’s parity, child’s Aboriginality, whether child speaks English as a second language, highest level of maternal school education, highest level of occupation of either parent, area-level disadvantage, and geographical remoteness, antenatal care visit before 20 weeks gestation, smoking during pregnancy, and preschool/day care attendance in the year before school. AEDC, Australian Early Development Census.
(TIF)

S4 Fig The age- and sex-adjusted absolute risk of developmental vulnerability on ≥1 AEDC domains, by maternal age at childbirth, in children with available outcome and exposure data (black squares) and the study population for this analysis (i.e., children enrolled at NSW public schools) (medium grey circles). Black squares, estimates for children with available outcome and exposure data (*N* = 152,556); medium grey circles, estimates for the study population for this analysis (i.e., children enrolled at NSW public schools) (*N* = 98,918). AEDC, Australian Early Development Census; NSW, New South Wales.
(TIF)

S5 Fig The distribution of maternal age at childbirth for children in the study population, overlaid with the proportion of children who were developmentally vulnerable or had medically diagnosed special needs^1^, by maternal age at childbirth (*N* = 103,685^2^). (1) Includes 4,670 children who were medically diagnosed as having high needs requiring special assistance due to chronic medical, physical, or intellectually disabling conditions. (2) Although 104,200 children had complete data for maternal age and at least one outcome variable (as per S1 Fig), 515 children did not have data for the aggregate outcome (i.e., vulnerable on ≥1 AEDC domains); as such, the total number of children in this sensitivity analysis was 103,685. AEDC, Australian Early Development Census.
(TIF)

S6 Fig Comparison of results from piecewise linear regression models using imputed dataset (*N* = 99,432) versus complete case dataset (*N* = 78,229) to estimate the absolute risk of developmental vulnerability on ≥1 AEDC domain, by maternal age at childbirth. Model 1 includes adjustment for the child’s age at school entry, sex, and AEDC year; in addition to the covariates included in Model 1, Model 3 adjusts for private health insurance/patient status, mother born in Australia/overseas, mother partnered/single parent, mother’s parity, child’s Aboriginality, child speaks English as a second language, highest level of maternal school education, highest level of occupation of either parent, area-level disadvantage, geographical remoteness, antenatal care visit before 20 weeks gestation, smoking during pregnancy, and preschool/day care attendance in the year before school. AEDC, Australian Early Development Census.
(TIF)

S1 RECORD Checklist RECORD statement. (DOCX)

S1 Table Sociodemographic, perinatal, and early childhood characteristics from the (i) original data for 99,530 children in the study population, (ii) original data for 78,697 children with complete covariate information (i.e., complete cases) and (iii) imputed data for 99,530 children in the study population. AEDC, Australian Early Development Census.
(DOCX)

S2 Table Comparison of quadratic and piecewise linear parameterisations of maternal age at childbirth in regression models of developmental vulnerability on ≥1 AEDC domain, including the AIC for each model. AEDC, Australian Early Development Census; AIC, Akaike Information Criterion.
(DOCX)

S1 Text Summary of methods and findings for the comparison of results from nonlinear (quadratic) and piecewise linear regression models. (DOCX)

## Abbreviations

AEDC: Australian Early Development Census
AIC: Akaike Information Criterion
ARIA+: Accessibility/Remoteness Index of Australia
IQR: interquartile range
NSW: New South Wales
SD: standard deviation
