# Supplementary figures and images for: Maternal age and offspring developmental vulnerability at age five: A population-based cohort study of Australian children
Source: PLoS Med. 2018 Apr 24;15(4):e1002558. doi: 10.1371/journal.pmed.1002558 (PMC5915778; doi:10.1371/journal.pmed.1002558)

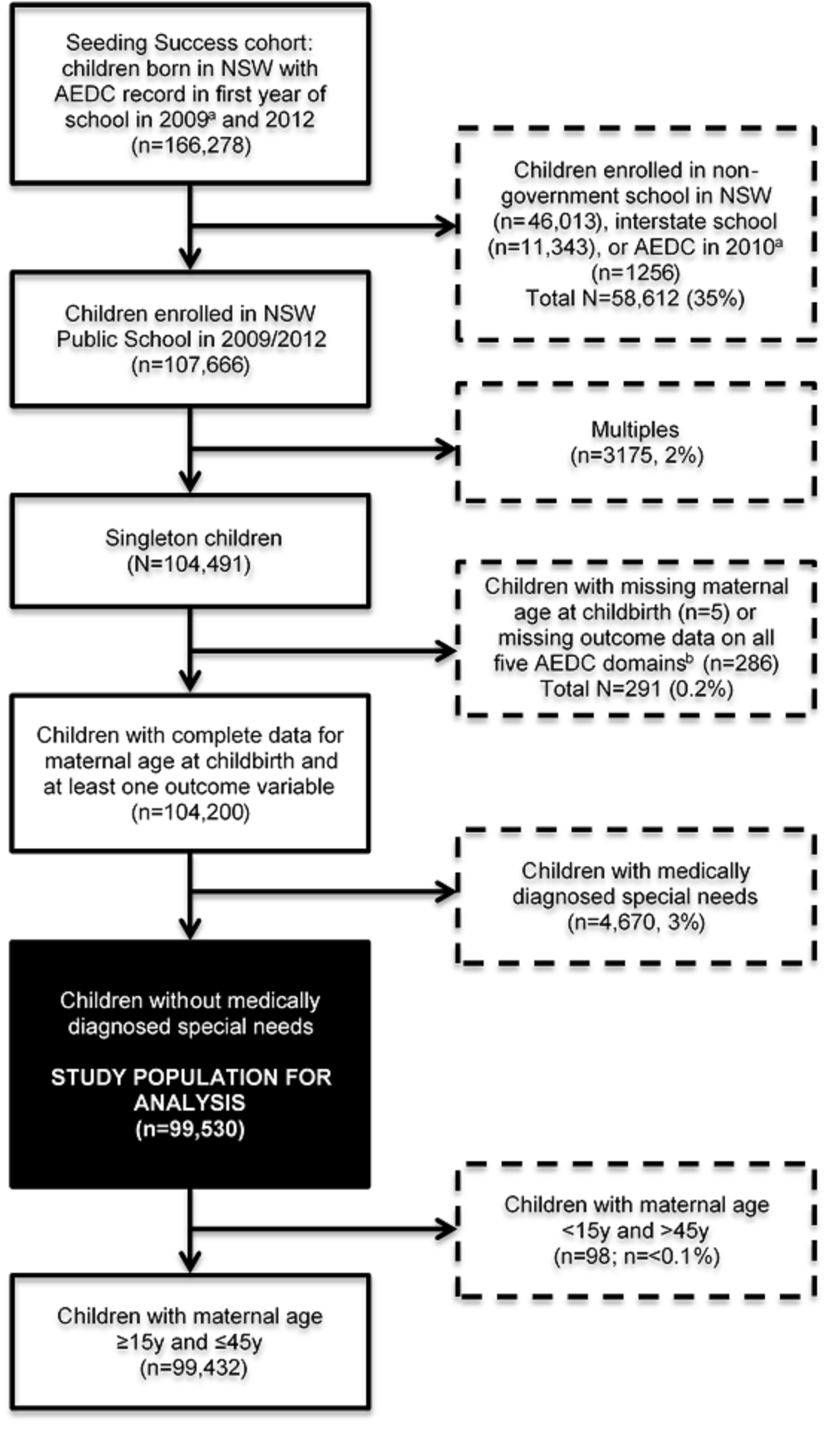

Supplement: S1 Fig — aIn 2010, additional AEDC data were collected in NSW to increase numbers of children in areas with small sample sizes in the 2009 AEDC. bThese children with missing outcome data (n = 286) did not include children with medically diagnosed special needs who were excluded in the following step. AEDC, Australian Early Development Census; NSW, New South Wales. (TIF) [file pmed.1002558.s001.tif]

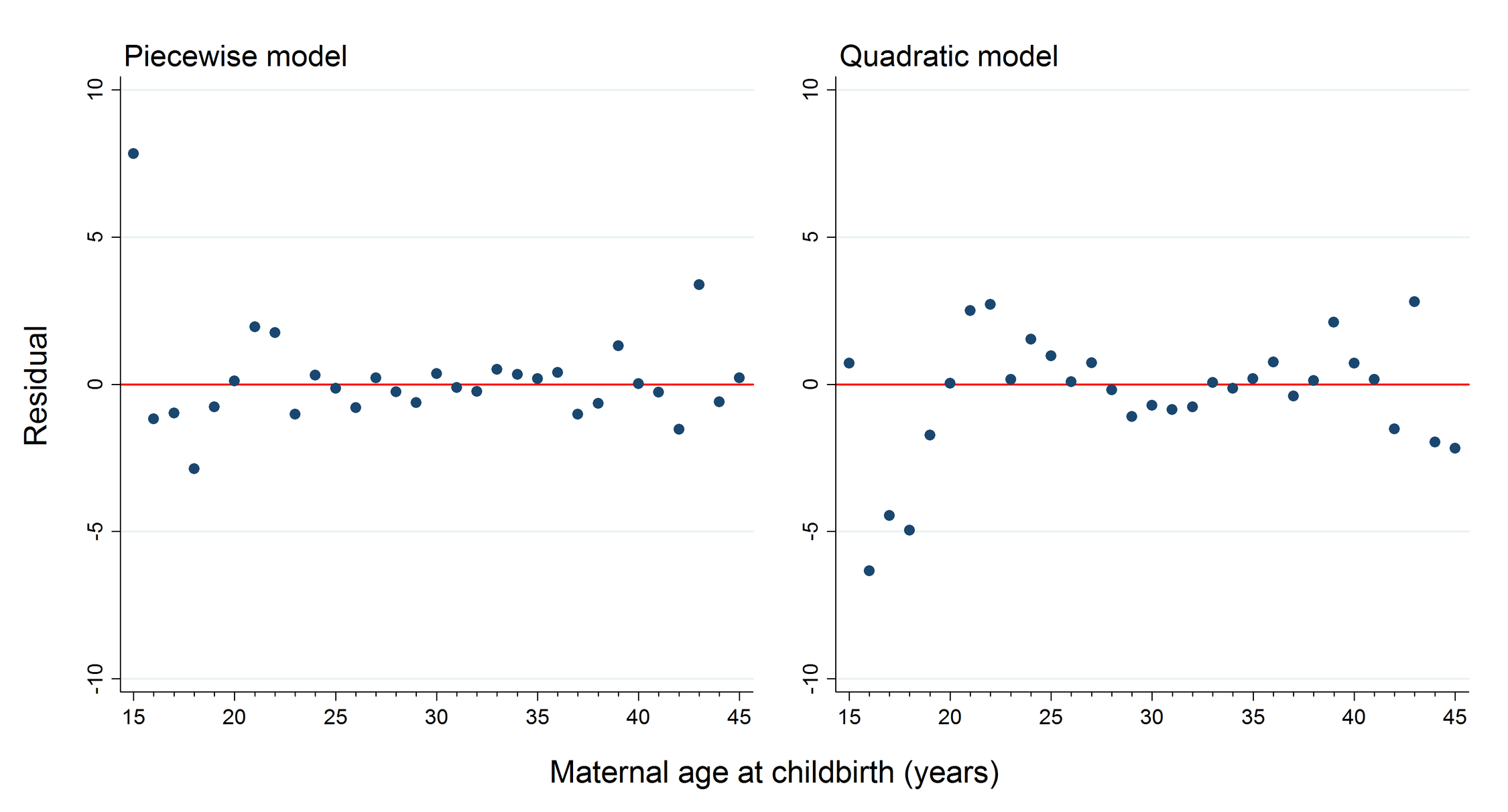

Supplement: S2 Fig — AEDC, Australian Early Development Census. (TIF) [file pmed.1002558.s002.tif]

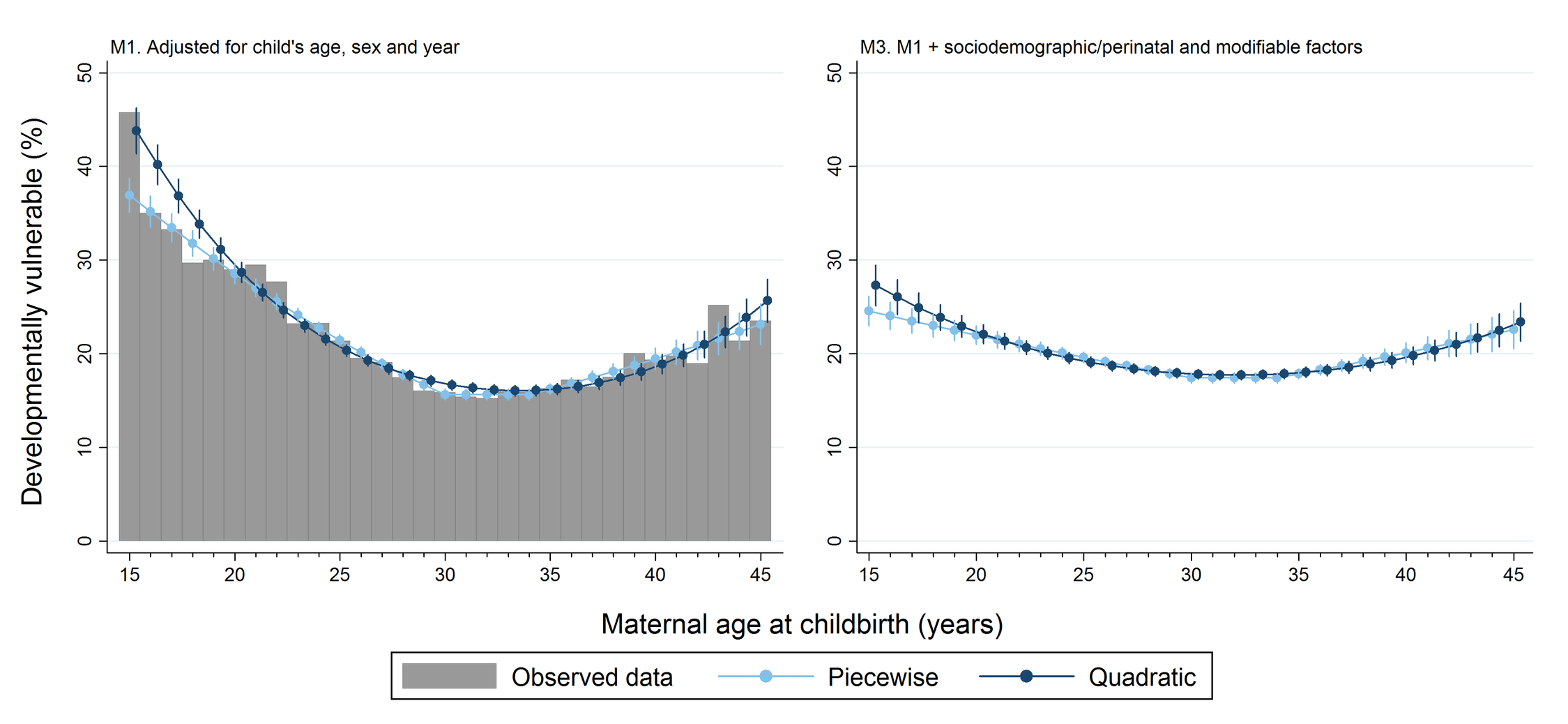

Supplement: S3 Fig — Reference group for quadratic models, maternal age at childbirth of 30 years. Model 1 includes adjustment for the child’s age at school entry, sex, and AEDC year; in addition to the covariates included in Model 1, Model 3 adjusts for private health insurance/patient status, mother born in Australia/overseas, mother partnered/single parent, mother’s parity, child’s Aboriginality, whether child speaks English as a second language, highest level of maternal school education, highest level of occupation of either parent, area-level disadvantage, and geographical remoteness, antenatal care visit before 20 weeks gestation, smoking during pregnancy, and preschool/day care attendance in the year before school. AEDC, Australian Early Development Census. (TIF) [file pmed.1002558.s003.tif]

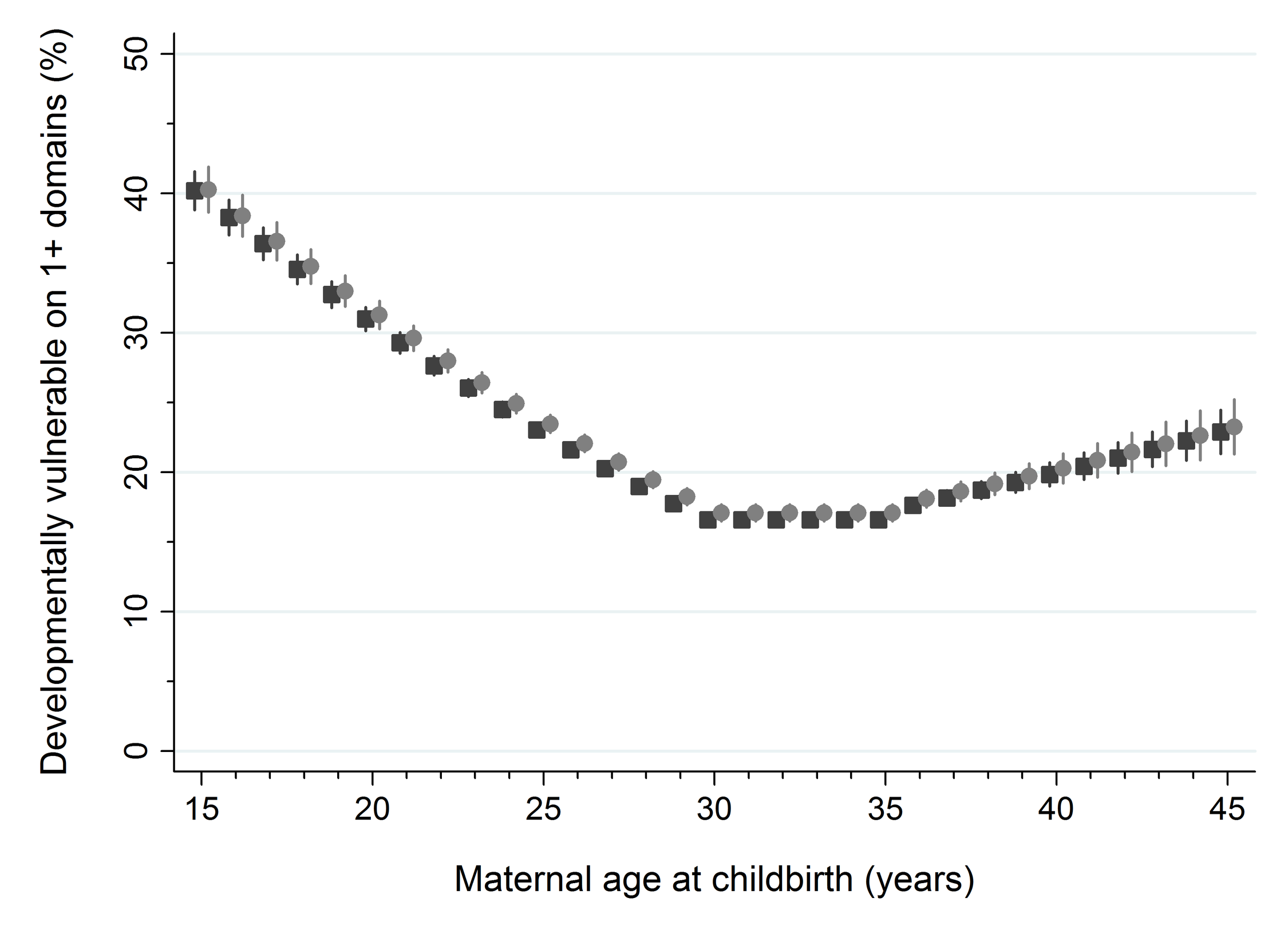

Supplement: S4 Fig — Black squares, estimates for children with available outcome and exposure data (N = 152,556); medium grey circles, estimates for the study population for this analysis (i.e., children enrolled at NSW public schools) (N = 98,918). AEDC, Australian Early Development Census; NSW, New South Wales. (TIF) [file pmed.1002558.s004.tif]

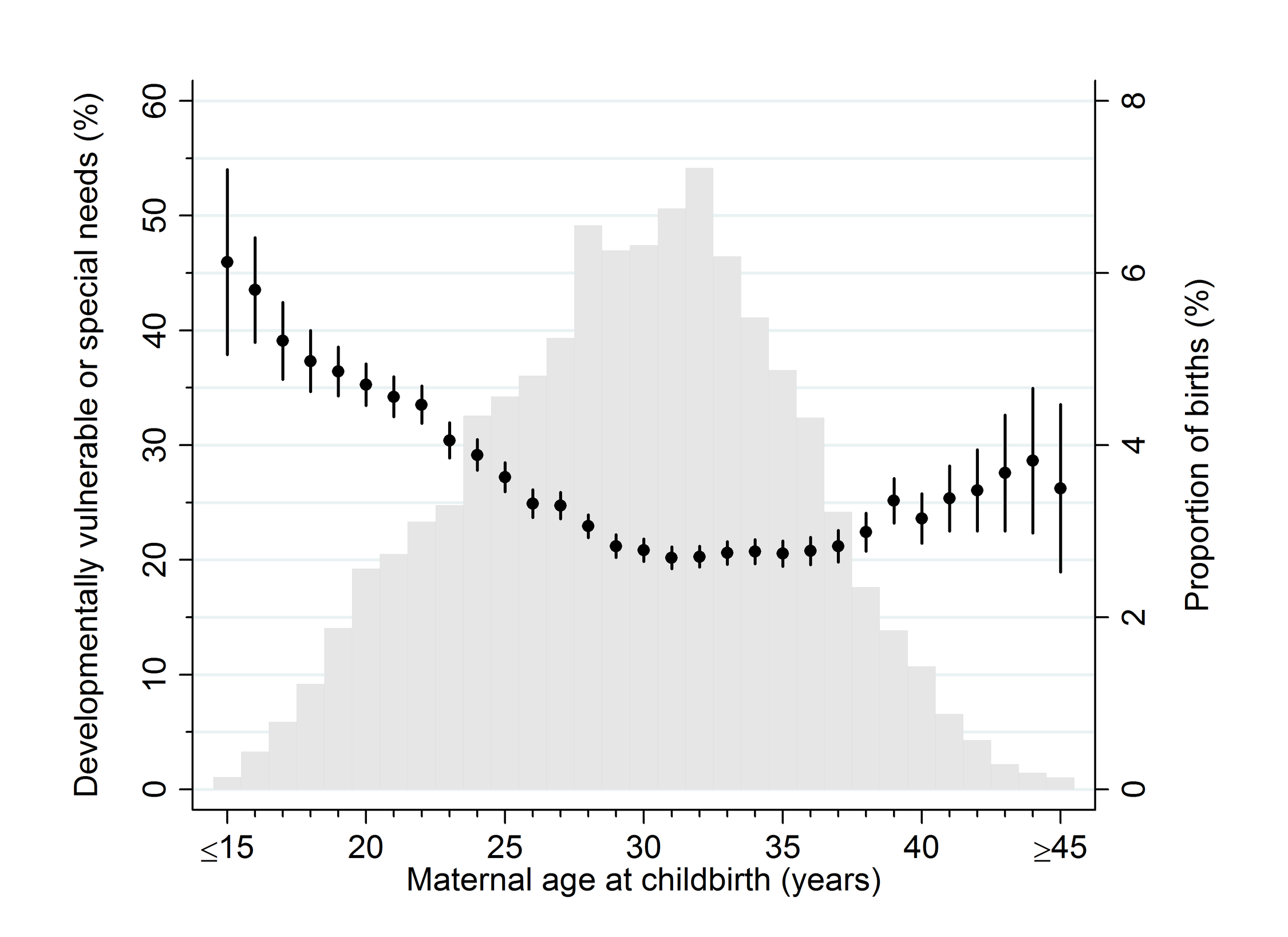

Supplement: S5 Fig — (1) Includes 4,670 children who were medically diagnosed as having high needs requiring special assistance due to chronic medical, physical, or intellectually disabling conditions. (2) Although 104,200 children had complete data for maternal age and at least one outcome variable (as per S1 Fig), 515 children did not have data for the aggregate outcome (i.e., vulnerable on ≥1 AEDC domains); as such, the total number of children in this sensitivity analysis was 103,685. AEDC, Australian Early Development Census. (TIF) [file pmed.1002558.s005.tif]

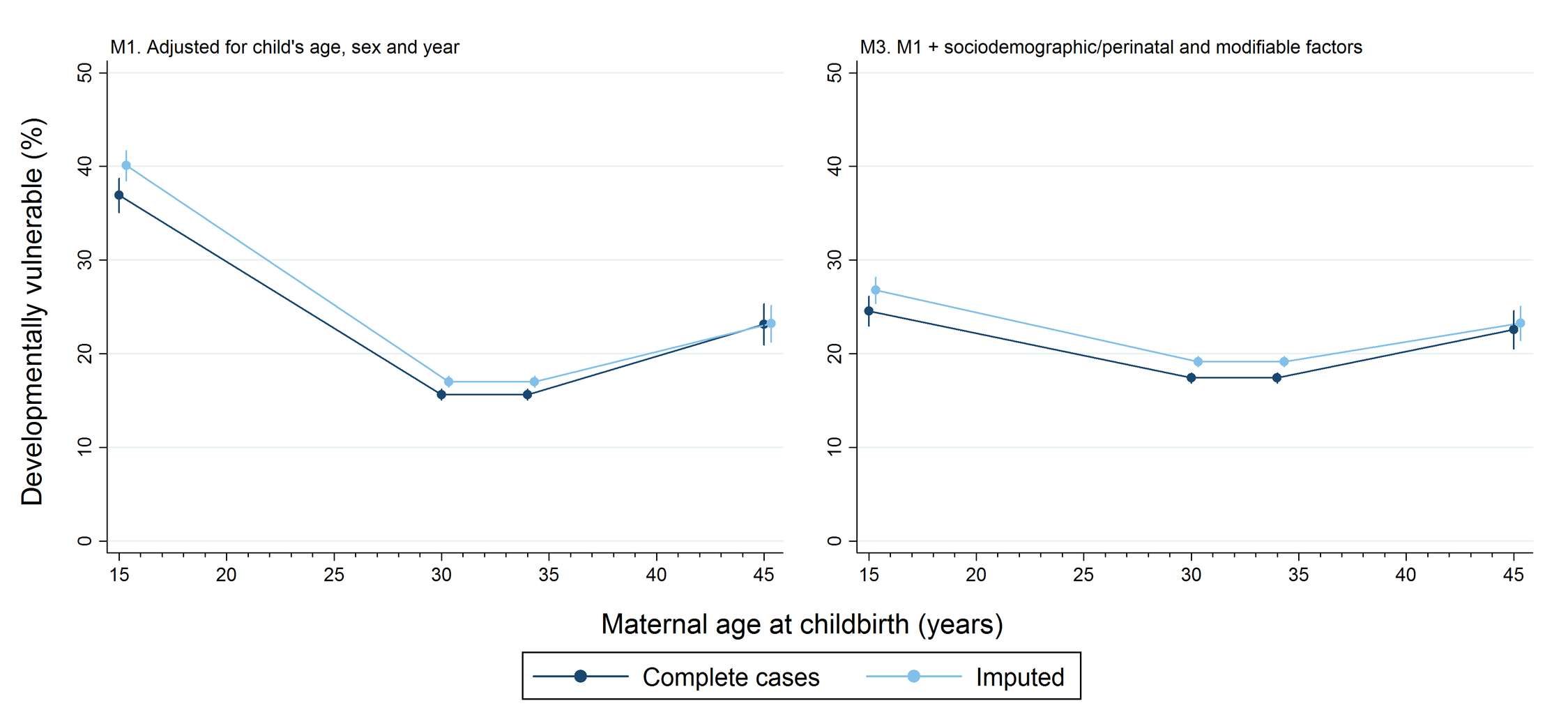

Supplement: S6 Fig — Model 1 includes adjustment for the child’s age at school entry, sex, and AEDC year; in addition to the covariates included in Model 1, Model 3 adjusts for private health insurance/patient status, mother born in Australia/overseas, mother partnered/single parent, mother’s parity, child’s Aboriginality, child speaks English as a second language, highest level of maternal school education, highest level of occupation of either parent, area-level disadvantage, geographical remoteness, antenatal care visit before 20 weeks gestation, smoking during pregnancy, and preschool/day care attendance in the year before school. AEDC, Australian Early Development Census. (TIF) [file pmed.1002558.s006.tif]
