# Supplementary material for: Maternal age and offspring developmental vulnerability at age five: A population-based cohort study of Australian children
Source: PLoS Med. 2018 Apr 24;15(4):e1002558. doi: 10.1371/journal.pmed.1002558 (PMC5915778; doi:10.1371/journal.pmed.1002558)
Supplement: S1 Table — AEDC, Australian Early Development Census. (DOCX) [file pmed.1002558.s008.docx]

S1 Table. Socio-demographic, perinatal and early childhood characteristics from the: (i) original data for 99,530 children in the study population, (ii) original data for 78,697 children with complete covariate information (i.e. complete cases), and (iii) imputed data for 99,530 children in the study population.

|  | **Study population**  (99,530) | | | **Complete case sample**  (N=78,697) | | **Imputed sample**  (N=99,530) | |
| --- | --- | --- | --- | --- | --- | --- | --- |
|  | Total N observed | n | (%)^1^ | n | (%)^1^ | n | (%)^1^ |
| **Outcome: AEDC vulnerability** |  |  |  |  |  |  |  |
| Physical health and wellbeing domain | 99437 | 8887 | (8.9) | 6212 | (7.9) | 8901 | (8.9) |
| Social competence domain | 99380 | 8698 | (8.7) | 6092 | (7.7) | 8712 | (8.8) |
| Emotional maturity domain | 98935 | 6822 | (6.9) | 4852 | (6.2) | 6894 | (6.9) |
| Language and cognitive skills domain | 99434 | 5552 | (5.6) | 3698 | (4.7) | 5566 | (5.6) |
| Communication skills and general knowledge domain | 99428 | 8795 | (8.8) | 6221 | (7.9) | 8803 | (8.8) |
| One or more domains | 99015 | 20652 | (20.7) | 14790 | (18.8) | 20675 | (20.8) |
| **Exposure: maternal age at childbirth (years)** | 99530 |  |  |  |  |  |  |
| Mean (SD) |  | 29.6 | (5.7) | 29.8 | (5.5) | 29.6 | (5.7) |
| Median (IQR) |  | 30 | (26-34) | 30 | (26-34) | 30 | (26-34) |
| Range |  | 13-56 |  | 13-56 |  | 13-56 |  |
| **Socio-demographic characteristics at child’s birth** |  |  |  |  |  |  |  |
| Age at start of school (years), mean (SD) | 99530 | 5.2 | (0.3) | 5.2 | (0.3) | 5.2 | (0.3) |
| Female sex, n (%) | 99530 | 49040 | (49.3) | 38724 | (49.2) | 49040 | (49.3) |
| Aboriginal and/or Torres Strait Islander^2^, n (%) | 99530 | 7206 | (7.2) | 4490 | (5.7) | 7206 | (7.2) |
| Mother born in Australia^2^, n (%) | 99032 |  |  |  |  |  |  |
| No |  | 26625 | (26.8) | 20979 | (26.7) | 26751 | (26.9) |
| Yes |  | 72407 | (72.7) | 57718 | (73.3) | 72779 | (73.1) |
| Missing |  | 498 | (0.5) | - | - | - | - |
| Mother married/partnered^3^, n (%) | 96440 |  |  |  |  |  |  |
| No |  | 18271 | (18.4) | 12841 | (16.3) | 19209 | (19.3) |
| Yes |  | 78169 | (78.5) | 65856 | (83.7) | 80321 | (80.7) |
| Missing |  | 3090 | (3.1) | - | - | - | - |
| Private health insurance/patient^3^, n (%) | 98491 |  |  |  |  |  |  |
| No |  | 67868 | (68.2) | 52218 | (66.4) | 68614 | (68.9) |
| Yes |  | 30623 | (30.8) | 26479 | (33.6) | 30916 | (31.1) |
| Missing |  | 1039 | (1.0) | - | - | - | - |
| Geographical remoteness of residence^4^, n (%) | 98316 |  |  |  |  |  |  |
| Major city |  | 62978 | (63.3) | 50674 | (64.4) | 63718 | (64.0) |
| Inner regional area |  | 25481 | (25.6) | 20502 | (26.1) | 25829 | (26.0) |
| Outer regional area |  | 8900 | (8.9) | 6877 | (8.7) | 9012 | (9.1) |
| Remote/very remote area |  | 957 | (1.0) | 644 | (0.8) | 971 | (1.0) |
| Missing |  | 1214 | (1.2) | - | - | - | - |
| Area-level disadvantage^4^, n (%) | 98316 |  |  |  |  |  |  |
| Quintile 1 (Most disadvantaged) |  | 9457 | (9.5) | 6874 | (8.7) | 9575 | (9.6) |
| Quintile 2 |  | 11026 | (11.1) | 8594 | (10.9) | 11171 | (11.2) |
| Quintile 3 |  | 34373 | (34.5) | 26914 | (34.2) | 34801 | (35.0) |
| Quintile 4 |  | 20261 | (20.4) | 16651 | (21.2) | 20504 | (20.6) |
| Quintile 5 (Least disadvantaged) |  | 23199 | (23.3) | 19664 | (25.0) | 23479 | (23.6) |
| Missing |  | 1214 | (1.2) | - | - | - | - |
| **Pregnancy characteristics relating to child’s birth** |  |  |  |  |  |  |  |
| Mother’s number of prior births, n (%) | 98142 |  |  |  |  |  |  |
| No prior births, n (%) |  | 40143 | (40.3) | 32433 | (41.2) | 40691 | (40.9) |
| One prior birth, n (%) |  | 33661 | (33.8) | 27768 | (35.3) | 34120 | (34.3) |
| Two or more prior births, n (%) |  | 24338 | (24.5) | 18496 | (23.5) | 24719 | (24.8) |
| Missing |  | 1388 | (1.4) | - | - | - | - |
| Antenatal care before 20 weeks gestation, n (%) | 97289 |  |  |  |  |  |  |
| No |  | 11056 | (11.1) | 7917 | (10.1) | 11369 | (11.4) |
| Yes |  | 86233 | (86.6) | 70780 | (89.9) | 88161 | (88.6) |
| Missing |  | 2241 | (2.3) | - | - | - | - |
| Mother smoked during pregnancy, n (%) | 97833 |  |  |  |  |  |  |
| No |  | 81567 | (82.0) | 67775 | (86.1) | 82981 | (83.4) |
| Yes |  | 16266 | (16.3) | 10922 | (13.9) | 16549 | (16.6) |
| Missing |  | 1697 | (1.7) | - | - | - | - |
| **Socio-demographic and other characteristics measured in child’s first year at school** | | | |  |  |  |  |
| AEDC year: 2009 | 99530 | 47083 | (47.3) | 37899 | (48.2) | 47083 | (47.3) |
| AEDC year: 2012 |  | 52447 | (52.7) | 40798 | (51.8) | 52447 | (52.7) |
| Preschool/childcare in year before school, n (%) | 93058 |  |  |  |  |  |  |
| No |  | 10618 | (10.7) | 8329 | (10.6) | 11645 | (11.7) |
| Yes |  | 82440 | (82.8) | 70368 | (89.4) | 87885 | (88.3) |
| Missing |  | 6472 | (6.5) | - | - | - | - |
| English second language, n (%) | 99530 | 16914 | (17.0) | 13043 | (16.6) | 16914 | (17.0) |
| Mother’s highest level of school education, n (%) | 91015 |  |  |  |  |  |  |
| Year 12^5^, n (%) |  | 57360 | (57.6) | 50583 | (64.3) | 61494 | (61.8) |
| Year 11, n (%) |  | 6754 | (6.8) | 5644 | (7.2) | 7580 | (7.6) |
| Year 10, n (%) |  | 21304 | (21.4) | 18110 | (23.0) | 23940 | (24.1) |
| ≤Year 9, n (%) |  | 5597 | (5.6) | 4360 | (5.5) | 6516 | (6.5) |
| Missing |  | 8515 | (8.6) | - | - | - | - |
| Highest level occupation of either parent^6^, n (%) | 92698 |  |  |  |  |  |  |
| Managers/professionals |  | 23542 | (23.7) | 20816 | (26.5) | 24662 | (24.8) |
| Business managers/associate professionals |  | 22016 | (22.1) | 19371 | (24.6) | 23259 | (23.4) |
| Trades/clerks/services |  | 23165 | (23.3) | 19955 | (25.4) | 24878 | (25.0) |
| Drivers/hospitality/labourers |  | 15621 | (15.7) | 12436 | (15.8) | 17190 | (17.3) |
| Not in paid work in last 12 months |  | 8354 | (8.4) | 6119 | (7.8) | 9541 | (9.6) |
| Missing |  | 6832 | (6.9) | - | - | - | - |

AEDC, Australian Early Development Census. 1. n(%) unless other statistic reported in left hand table column. 2. Defined as child or parent identified as Aboriginal on any of the birth records (i.e. perinatal data collection, birth registration or hospital birth record), or AEDC school record; 3. Based on hospital birth record; 4. Based on mother’s statistical local area of residence recorded in the Perinatal Data Collection; 5. Highest level of school education in Australia; 6. Based on highest ranking occupation of either parent recorded on school enrolment.
