# Supplementary material for: Maternal age and offspring developmental vulnerability at age five: A population-based cohort study of Australian children
Source: PLoS Med. 2018 Apr 24;15(4):e1002558. doi: 10.1371/journal.pmed.1002558 (PMC5915778; doi:10.1371/journal.pmed.1002558)
Supplement: S2 Table — AEDC, Australian Early Development Census; AIC, Akaike Information Criterion. (DOCX) [file pmed.1002558.s009.docx]

S2 Table. Comparison of quadratic and piecewise linear parameterisations of maternal age at childbirth in regression models of developmental vulnerability on ≥1 AEDC domain, including the Akaike Information Criterion (AIC) for each model.

| Model specification of maternal age | Covariate adjustment | Number of parameters for maternal age parameterisation | AIC |
| --- | --- | --- | --- |
| Quadratic | No | 2 | 74963.4 |
| Piecewise linear (knots at 30 and 35 years) | No | 2 | 74927.9 |
| Piecewise linear (knots at 20, 30 and 35 years) | No | 3 | 74927.4 |
| Quadratic | Yes^a^ | 2 | 69441.7 |
| Piecewise linear (knots at 30 and 35 years) | Yes^a^ | 2 | 69419.7 |
| Piecewise linear (knots at 20, 30 and 35 years) | Yes^a^ | 3 | 69419.8 |

AIC, Akaike Information Criterion. ^a^Includes adjustment for the child’s age at school entry, sex and AEDC year, private health insurance/patient status, mother born in Australia/overseas, mother partnered/single parent, mother’s parity, child’s Aboriginality, child speaks English as a second language, highest level of maternal school education, highest level of occupation of either parent, area-level disadvantage and geographical remoteness antenatal care visit before 20 weeks gestation, smoking during pregnancy, and preschool/daycare attendance in the year before school.
