# Supplementary material for: Maternal age and offspring developmental vulnerability at age five: A population-based cohort study of Australian children
Source: PLoS Med. 2018 Apr 24;15(4):e1002558. doi: 10.1371/journal.pmed.1002558 (PMC5915778; doi:10.1371/journal.pmed.1002558)
Supplement: S1 Text — (DOCX) [file pmed.1002558.s010.docx]

**S1 Text: Summary of methods and findings for the comparison of results from non-linear (quadratic) and piece-wise linear regression models.**

In the analysis presented in our original manuscript submission, we modelled the risk of developmental vulnerability as a quadratic function of maternal age, to capture the observed reverse J-shaped pattern of risk. Following peer review, we explored piecewise linear parameterisation of maternal age to determine whether this was a better fit to the data than the original quadratic models. We explored two specifications of the piecewise model: the first divided the maternal age range into three segments (i.e. 15-<30 years, 30-<35 years, ≥35-45 years), the second divided maternal age into four segments (i.e. 15-<20 years, 20-<30 years, 30-<35 years, ≥35-45 years). These specifications were based on the patterns observed in the raw data and common 5-year cut-points used to categorise maternal age at childbirth in the related literature. For each piecewise linear regression model, the slope of the line between ages 30-<35 was constrained to zero, as suggested in peer review. Model fit was compared using the Akaike Information Criterion (AIC).

We found there was a small improvement in the model fit using piecewise linear regression models compared with the quadratic models, as evidenced by a reduction in the AIC statistic (S2 Table). Comparing the two piecewise models, the improvement in model fit when specifying four segments compared with three segments did not compensate for the inclusion of an additional parameter, i.e. there was no reduction in the AIC between these two models. Based on this, we proceeded to apply the simpler piecewise linear regression model (i.e. three segments) for the main analysis of the revised manuscript.

Examining residual plots for the piecewise versus quadratic models suggested that the main improvements in model fit were at the younger extreme of maternal age (S2 Fig). To further assess differences in the use of the piecewise versus quadratic parameterisation of maternal age, we calculated the estimates of absolute risk, risk difference and relative risk for Model 1 (i.e. age and sex adjusted models described in the main analysis methods) and Model 3 (i.e. Model 1 plus further adjustment for socio-demographic and modifiable factors, as described in the main analysis methods). Consistent with above, S3 Fig shows that the piecewise linear specification better captures the crude relationship between maternal age and developmental vulnerability at the younger extreme of maternal age; however, the differences between models were negligible in Model 3.

Based on the above findings, we revised the manuscript to present the results from the piecewise linear regression model, and noted in the revised manuscript that we modified the methods based on peer review.
